# Editorial: Beyond abstinence: harm reduction and its impact on addiction disorders

**DOI:** 10.3389/fpsyt.2026.1898442

**Published:** 2026-06-26

**Authors:** Miesha Marzell

**Affiliations:** Division of Public Health, Decker College of Nursing and Health Sciences, Binghamton University, Binghamton, NY, United States

**Keywords:** addiction treatment, harm reduction, health equity, public health, substance use disorders

Harm reduction has emerged as a critical public health framework for addressing the complex health, social, and structural challenges associated with substance use. While traditional approaches have often emphasized abstinence as the primary marker of success, contemporary harm reduction strategies recognize that reducing risk, improving quality of life, increasing treatment engagement, and promoting health equity are equally important outcomes. This Research Topic, *“Beyond Abstinence: Harm Reduction and Its Impact on Addiction Disorders,”* sought to advance understanding of innovative, evidence-based, and community-centered approaches that reduce the adverse consequences associated with substance use and addiction.

The contributions included in this Research Topic illustrate the breadth of contemporary harm reduction practice, spanning clinical treatment, psychosocial intervention, and service innovation. Collectively, these articles demonstrate that effective harm reduction extends beyond any single intervention and instead represents a continuum of supports designed to meet individuals where they are while reducing harms and promoting wellbeing.

In a large national analysis of treatment outcomes for opioid use disorder, Graziane examined the real-world effectiveness of medication-assisted treatment and psychotherapy across multiple healthcare systems. The findings demonstrated that both medication for opioid use disorder and structured psychotherapy were independently associated with improved remission outcomes, while integrated treatment approaches produced the strongest effects. These results reinforce longstanding evidence that comprehensive, multimodal approaches are essential for addressing the complexity of opioid use disorder and highlight the value of integrating behavioral health supports within routine addiction care.

Extending the discussion to cannabis use, Giguère et al. conducted a systematic review and meta-analysis examining psychological interventions delivered by health professionals in community settings. Their findings suggest that cognitive behavioral therapy and motivational approaches may offer modest benefits in reducing cannabis-related problems and the severity of dependence. At the same time, the review highlights important gaps in the evidence base and underscores the need for future research focused on meaningful health and functional outcomes rather than consumption patterns alone. These findings align closely with harm reduction principles by emphasizing improvements in well-being and functioning as important indicators of success.

At the policy and service delivery level, James et al. described the implementation of a novel needle and syringe program initiative supporting individuals who inject methadone in New South Wales, Australia. Following policy changes that permitted the distribution of specialized injection equipment, the program demonstrated high acceptability among participants and reported improvements in safety and health outcomes. This work illustrates how responsive policy reform and community-informed service innovation can address longstanding inequities in access to harm reduction resources for populations whose needs have often been overlooked.

An additional theme emerging from this Research Topic is the recognition that harm reduction extends beyond individual behavior change and immediate risk mitigation. Contemporary harm reduction frameworks increasingly acknowledge the importance of addressing the social, structural, and service environments that shape vulnerability and health outcomes. While the articles in this Research Topic examine different populations and intervention approaches, each reflects a broader commitment to meeting individuals where they are and reducing harms through accessible, evidence-informed, and responsive systems of care. Collectively, these contributions remind us that substance-related harms are not produced solely by individual behaviors but are also shaped by social, policy, and healthcare environments that influence vulnerability, access to care, and opportunities for recovery. This perspective encourages researchers, practitioners, and policymakers to consider not only how harms are reduced but also how vulnerabilities are created and sustained within healthcare, policy, and social systems.

Taken together, these contributions highlight several important themes. First, harm reduction operates across multiple levels, including clinical treatment, psychosocial support, public policy, and community-based services. Second, meaningful outcomes extend beyond abstinence and include reductions in risk, improvements in health and functioning, treatment engagement, and enhanced quality of life. Finally, the Research Topic reinforces the importance of tailoring interventions to the needs of diverse populations and recognizing that effective harm reduction requires flexibility, accessibility, and responsiveness to lived experience.

Although the Research Topic includes a limited number of contributions, the articles collectively demonstrate the continuing evolution of harm reduction as a pragmatic, evidence-informed public health approach, the articles collectively demonstrate the continuing evolution of harm reduction as a pragmatic, evidence-informed public health approach. Future research should continue to explore innovative interventions, strengthen the evidence base for emerging practices, and evaluate policies that expand equitable access to care. As substance use landscapes continue to evolve globally, harm reduction remains an essential framework for promoting health, dignity, equity, and social inclusion among people who use substances.

The editors thank all authors, reviewers, and contributors who participated in this Research Topic and helped advance the growing body of scholarship supporting harm reduction approaches to addiction and substance use.

